# TCR–like antibodies mediate complement and antibody-dependent cellular cytotoxicity against Epstein-Barr virus–transformed B lymphoblastoid cells expressing different HLA-A*02 microvariants

**DOI:** 10.1038/s41598-017-10265-6

**Published:** 2017-08-30

**Authors:** Junyun Lai, Joanna Ai Ling Choo, Wei Jian Tan, Chien Tei Too, Min Zin Oo, Manuel A. Suter, Fatimah Bte Mustafa, Nalini Srinivasan, Conrad En Zuo Chan, Andrew Guo Xian Lim, Youjia Zhong, Soh Ha Chan, Brendon J. Hanson, Nicholas R. J. Gascoigne, Paul A. MacAry

**Affiliations:** 1 Immunology Programme, Life Sciences Institute, National University of Singapore, Singapore, 117456 Singapore; 2 Department of Microbiology and Immunology, Yong Loo Lin School of Medicine, National University of Singapore, Singapore, 117545 Singapore; 3 Defense Medical and Environmental Research Institute, DSO National Laboratories, Singapore, 117510 Singapore

## Abstract

Epstein-Barr virus (EBV) is a common gammaherpesvirus associated with various human malignancies. Antibodies with T cell receptor-like specificities (TCR-like mAbs) provide a means to target intracellular tumor- or virus-associated antigens by recognising their processed peptides presented on major histocompatibility complex (MHC) class I (pMHC) complexes. These antibodies are however thought to be relevant only for a single HLA allele. Here, we show that HLA-A*02:01-restricted EBV antigenic peptides EBNA1_562-570_, LMP1_125-133_ and LMP2A_426-434_ display binding degeneracy towards HLA-A*02 allelic microvariants, and that these pMHC complexes are recognised by anti-EBV TCR-like mAbs E1, L1 and L2 raised in the context of HLA-A*02:01. These antibodies bound endogenously derived pMHC targets on EBV–transformed human B lymphoblastoid cell lines expressing A*02:01, A*02:03, A*02:06 and A*02:07 alleles. More importantly, these TCR-like mAbs mediated both complement-dependent and antibody-dependent cellular cytotoxicity of these cell lines *in vitro*. This finding suggests the utility of TCR-like mAbs against target cells of closely related HLA subtypes, and the potential applicability of similar reagents within populations of diverse HLA-A*02 alleles.

## Introduction

Major histocompatibility complex (MHC) class I presentation of peptides to T cell receptors (TCR) on T lymphocytes enables the immunosurveillance of the intracellular proteome of all nucleated cells. The display of tumor- or virus-associated antigenic peptides distinguishes malignant or infected cells from their healthy counterparts, and forms the basis by which aberrant cells are marked for recognition and killing. These characteristic peptide-MHC (pMHC) complexes therefore represent attractive candidates for targeted strategies that may minimise damage against healthy tissues during the treatment of cancer or virus-associated malignancies^1^. TCR-like monoclonal antibodies (TCR-like mAbs), which recognise a specific peptide in association with an MHC molecule (human leukocyte antigen, HLA in humans), permit the targeting of such inaccessible nuclear or cytoplasmic tumor- or virus-associated antigens based on binding to their MHC-presented peptides found on the surface of target cells. To date, TCR-like mAbs have been raised against peptides from many of such antigens^2–4^, and several of these antibodies have demonstrated efficacy against tumors in preclinical models. Similar to conventional therapeutic antibodies, TCR-like mAbs have also been shown to perform effector functions including direct killing^3, 5^, complement-dependent cytotoxicity (CDC)^6^, antibody-dependent cellular cytotoxicity (ADCC)^2^ and antibody-dependent phagocytosis^7^. One major limitation of TCR-like mAbs and other TCR recognition-based strategies in their potential clinical application however, is that these approaches are traditionally thought to provide coverage only towards a single HLA allele^8^.

HLAs are polygenic and highly polymorphic in nature. The diversity of HLA is believed to reflect an evolutionary selection for disease-protective alleles in the context of rapidly mutating pathogens^9^. There are more than ten subtypes within the most extensively studied HLA-A*02 alone, and their frequencies and distribution differ widely across populations^10, 11^. Nonetheless, binding degeneracy of peptides derived from several pathogens has been observed not only between HLA allelic microvariants^12–14^ but also across HLA loci^15, 16^. The capacity to trigger T cell responses may however differ between peptides and alleles in question^14, 15, 17–20^. Reagents that recognise the same peptide presented by closely related HLA variants may therefore be useful candidates for further development^8^.

Epstein-Barr virus (EBV) was the first human tumor virus discovered and it is estimated that more than 90% of the world’s population carries an asymptomatic form of the infection. EBV infects cells of both lymphoid and epithelial origin, and the virus has been associated with several human malignancies such as post-transplant lymphoproliferative disease, Burkitt’s lymphoma, Hodgkin’s lymphoma and nasopharyngeal carcinoma. EBV latent proteins that are commonly found in EBV-associated tumors include EBV nuclear antigen 1 (EBNA1), latent membrane proteins (LMP) 1 and 2A^21^. We previously described three anti-EBV TCR-like mAbs E1, L1 and L2 respectively targeting EBV antigenic peptides EBNA1_562-570_, LMP1_125-133_ and LMP2A_426-434_ presented on HLA-A*02:01. These antibodies detected their endogenous targets on EBV-positive cell lines, splenic lesions of EBV-infected humanized mice, as well as clinically relevant EBV-positive nasopharyngeal carcinoma biopsies^22^. These anti-EBV TCR-like mAbs further demonstrated anti-tumor activities *in vivo* ^7^, underscoring their potential utility against EBV-associated malignancies.

Here, we show that HLA-A*02:01-restricted EBV peptides EBNA1_562-570_, LMP1_125-133_ and LMP2A_426-434_ display binding degeneracy towards closely related A*02:03, A*02:06 and A*02:07 alleles that are commonly found in Asian populations, and that these pMHC complexes can be recognised by our anti-EBV TCR-like mAbs. We then engineered the antibodies into chimeric IgG1, and further demonstrate that these TCR-like mAbs can induce both CDC and ADCC against EBV-transformed B lymphoblastoid cell lines (BLCLs) of different HLA*A-02 subtypes. These results suggest that TCR-like mAbs and similar reagents which have been originally raised in the context of HLA-A*02:01 may functionally cross-react and provide additional coverage towards other HLA-A*02 microvariants.

## Materials and Methods

### HLA frequencies and polymorphisms

HLA-A and HLA-A*02 allele frequencies within the European, North American, Northeast Asian and Southeast Asian populations were calculated based on values obtained from the NCBI dbMHC database (http://www.ncbi.nlm.nih.gov/gv/mhc/). Alleles with more than 5% frequencies from each of the four populations were depicted. Peptide binding pockets and HLA-A*02 subtype polymorphic residues were highlighted using PyMOL (The PyMOL Molecular Graphics System, v1.8, Schrödinger, LLC) based on the structure of HCV NS3_1073-1081_ peptide-bound HLA-A2 molecule (pdb 3MRG).

### *In silico* prediction

Binding predictions for EBV EBNA1 (uniprot identifier: PRO_0000116175), LMP1 (PRO_0000038310) and LMP2A (PRO_0000116280) epitopes against HLA*A02 alleles were performed using the online bioinformatics platform NetMHC (version 4.0) (http://www.cbs.dtu.dk/services/NetMHC-4.0/), that is based on the artificial neural network method^23, 24^. A percentile rank (rank %) of less than 0.5 indicates a strong binder (++), 0.5 to less than 2 a weak binder (+), and above 5 a non-binder (−).

### pMHC ELISA

Recombinant biotinylated HLA-A*02:01, A*02:03, A*02:06 and A*02:07 carrying conditional peptides were subjected to UV irradiation in the presence of EBV EBNA1_562-570_ (FMVFLQTHI), LMP1_125-133_ (YLLEMLWRL) or LMP2A_426-434_ (CLGGLLTMV) peptides to facilitate exchange^25–27^. Peptide-MHC (pMHC) complexes were then added into streptavidin-coated wells in quadruplicates. For stability ELISA, pMHC complexes were detected using HRP-conjugated anti-beta-2-microglobulin antibody (Abcam). For ELISA with the TCR-like mAbs, pMHC complexes were incubated with serially diluted antibodies followed by HRP-conjugated secondary antibody as previously described^7^. Substrate solution 2,2′-azino-bis(3-ethylbenzothiazoline-6-sulphonic acid) was then added and reactions were quenched using 0.1% sodium azide and 0.1 M citric acid. Absorbance values were measured (415 nm) and graphs were plotted using GraphPad Prism.

### Cells

Blood samples were obtained from HLA-typed^16^ healthy donors with informed consent (DSRB/E/2008/00293). All experiments were conducted in accordance with the guidelines and protocols approved by the Institutional Review Board of the National University of Singapore. EBV BLCLs were generated from healthy donor peripheral blood mononuclear cells (PBMCs) as previously described^7^. BLCLs, BJAB (ATCC 68673) and T2 cells (ATCC CRL-1992) were maintained in RPMI-1640 media supplemented with 10% FBS, 1% penicillin/streptomycin at 37 °C, 5% CO_2_ (R10).

### Peptide pulsing

Acid-stripped BLCLs or T2 cells were incubated in R10 with EBNA1_562-570_, LMP1_125-133_, LMP2A_426-434_, *Influenza* virus M1_58-66_ (GILGFVFTL), or cytomegalovirus pp65_495-503_ (NLVPMVATV) peptide (Mimotopes) for 3 hours at 37 °C before washing and processing for flow cytometry.

### Flow cytometry

Cells were stained with murine or chimeric TCR-like mAbs for 2 hours at 4 °C in PBS containing 3% FBS and 0.01% sodium azide. Cells were then washed and stained with AlexaFluor-488 conjugated goat anti-mouse IgG (H + L) or goat anti-human IgG (ThermoFisher) antibody for 1 hour. Samples were acquired on BD LSRFortessa (BD Biosciences) and data were analysed on FlowJo (TreeStar).

### Antibody engineering and production

RNA was isolated from the monoclonal hybridoma and converted into cDNA using Superscript III RT (ThermoFisher). To generate the chimeric antibodies, the V_H_ V_L_ domains of the original murine antibodies were amplified and cloned into a pTT5 expression vector respectively containing either the framework for the human IgG1 heavy constant chain (IGHG1 UniProtKB P01857) or the kappa light chain (IGKC UniProtKB P01834). Constructs were transfected into HEK293-6E cells via branched polyethylenimine (Sigma-Aldrich) and antibodies were purified from supernatant using Protein A beads (ThermoFisher).

### CDC assay

5 × 10^4^ cells were resuspended in serum-free media and incubated with antibodies for 1 hour at 37 °C. Baby rabbit complement (Cedarlane) was then added and the mixture was further incubated for 3 hours. Supernatant was collected for the measurement of LDH release (Promega). For cellular vitality assay, cells were additionally processed for staining with Sytox Green and C12-resaurzin according to manufacturer’s protocol (Life Technologies). Samples were acquired using Attune NxT flow cytometer (Thermo) and data were analysed using FlowJo. % Cytotoxicity was determined based on: ((experimental lysis − spontaneous lysis)/(maximum lysis − spontaneous lysis)) * 100.

### ADCC assay

2 × 10^4^ target cells were labeled with carboxyfluorescein succinimidyl ester (CFSE) and incubated with 10 μg/mL of the respective chimeric antibodies, isotype or no antibody control for 30 mins before the addition of fresh PBMCs to an E:T ratio of 20:1. Cells were co-cultured for 4 hours at 37 °C before staining with 7-aminoactinomycin D (7-AAD) for acquisition on BD LSRFortessa. Dead target cells were defined as CFSE^+^7-AAD^+^ population, and % cytotoxicity was determined accordingly.

### Statistical analysis

Statistical analyses were performed using GraphPad Prism applying unpaired student’s t-test. P-values of <0.05 were considered statistically significant.

## Results and Discussion

### Diversity of HLA-A*02 allelic variants in populations

**Figure 1:**
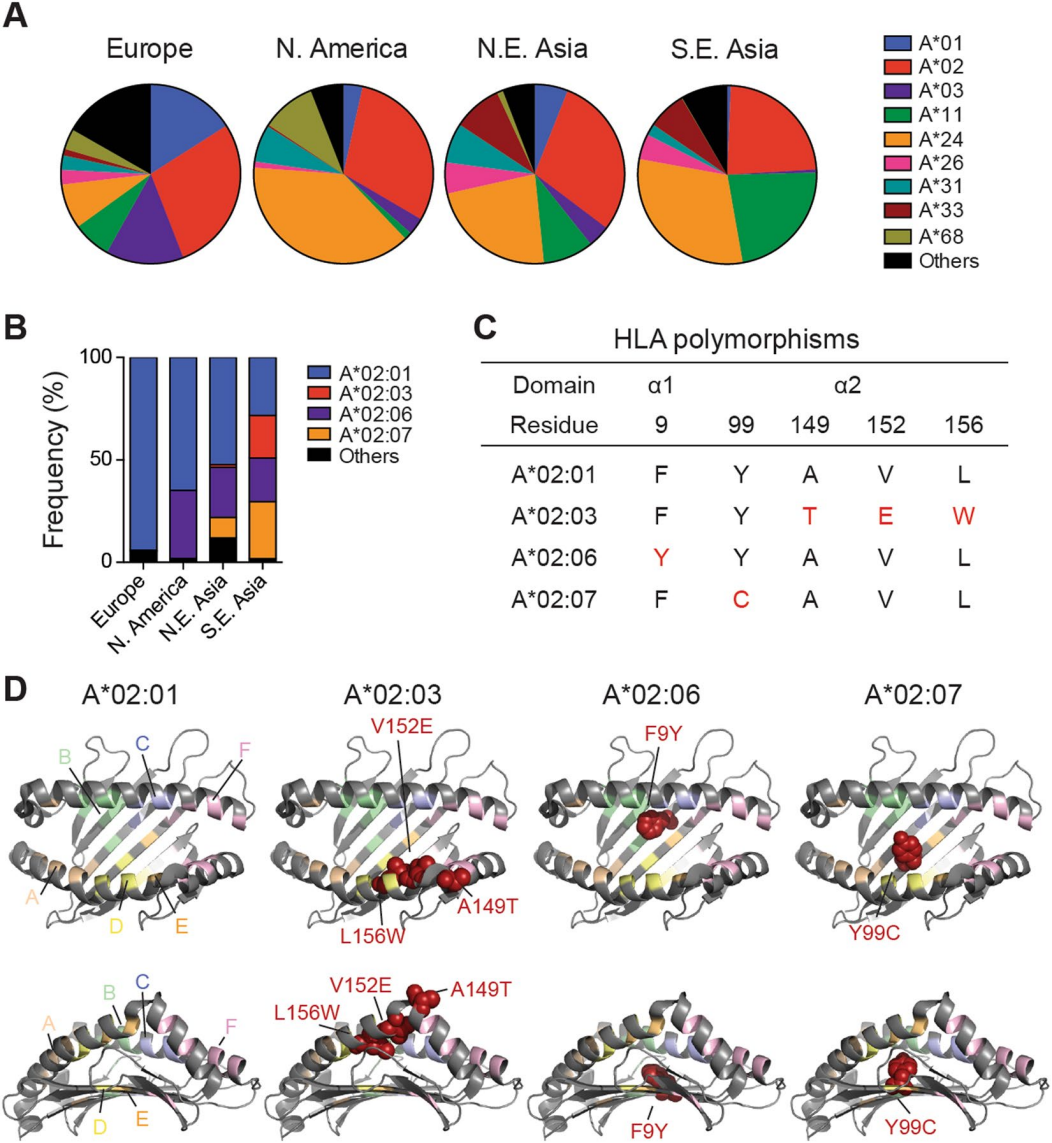
HLA-A*02 frequencies, distribution and alleles. (**A**) Frequencies of nine HLA-A loci within the European, North American, Northeast Asian and Southeast Asian populations. **(B)** HLA-A*02:01, A*02:03, A*02:06 and A*02:07 frequencies and distribution among the four populations. Alleles with frequencies of more than 5% from any of the four populations were illustrated. Values were extracted from the NCBI dbMHC database (http://www.ncbi.nlm.nih.gov/gv/mhc/). (**C**) Polymorphic amino acid residue changes between A*02:01, A*02:03, A*02:06 and A*02:07 found in the α1 and α2 domains of the MHC class molecule. (**D**) Top and side structural depiction of the four HLA-A*02 alleles, with peptide binding pockets highlighted and polymorphic residues indicated in red.

Among the HLA-A class I genes, HLA-A*02 represents one of the most prevalent alleles in the European (28.9%), North American (30.3%), Northeast Asian (29.3%) and Southeast Asian (24.5%) populations **(**Fig. 1A **)**. While A*02:01 is the predominant HLA-A*02 subtype found in all populations (Europeans – 94.1%, North Americans – 64.9%, Northeast Asian – 52.3%, Southeast Asian – 28.3%), other allelic variants including A*02:03 (Southeast Asian – 20.8%), A*02:06 (North Americans – 33.3%, Northeast Asian – 24.5%, Southeast Asian – 21.3%) and A*02:07 (Northeast Asian – 10.0%, Southeast Asian – 27.9%) are also expressed at high frequencies **(**Fig. 1B) (NCBI dbMHC database). Polymorphisms between these HLA-A*02 variants consist of amino acid substitutions **(**Fig. 1C **)** that are localised within the peptide binding groove of the MHC class I molecule **(**Fig. 1D **)**. As most of the TCR-like mAbs and TCR-engineered T cell strategies are focused on HLA-A*02:01 and its restricted antigens, the diversity of HLA-A*02 allelic variants highlights a need to evaluate whether such therapies would potentially be applicable to other closely related subtypes.

### HLA-A*02:01-restricted EBV peptides bind HLA-A*02 variants

**Figure 2:**
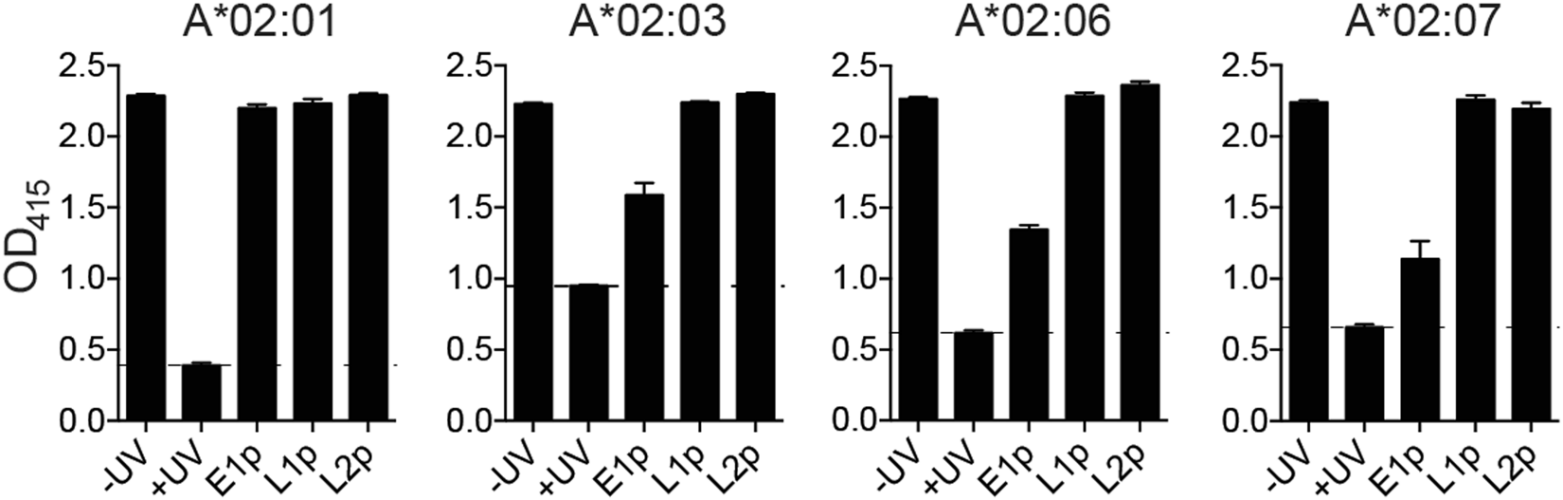
EBV peptides EBNA1_562-570_, LMP1_125-133_ and LMP2A_426-434_ bind HLA-A*02 allelic variants. Peptide-MHC stability ELISA was performed to determine the binding of HLA-A*02:01-restricted EBV peptides to A*02:03, A*02:06 and A*02:07. Biotinylated MHC complexes with UV-cleavable cognate peptides were exchanged in the presence of the three respective EBV peptides. Exchanged complexes were added onto streptavidin-coated ELISA wells and β2m, which non-covalently interacts with the MHC class I, was subsequently detected as an indication of the structural integrity of the whole complex. Refer to Supplementary Figure S1 for a schematic illustration of the methodology.

EBV EBNA1_562-570_, LMP1_125-133_ and LMP2A_426-434_ were originally identified as A*02:01-restricted binding peptides^19, 28, 29^. A*02:01, A*02:03, A*02:06 and A*02:07 are classified under the A2-supertype with preferences for small or aliphatic hydrophobic residues at position 2 and C-terminus of its binding peptide^30^. Therefore, to predict whether these three EBV peptides could bind allelic variants other than A*02:01, we performed computational analyses on NetMHC, which predicts peptide-MHC binding based on artificial neural networks^23, 24^. EBV peptides were predicted to bind HLA-A*02 subtypes with varying degrees, with the exception of EBNA1_562-570_ to A*02:07 **(**Table 1 **)**. Indeed, LMP1_125-133_–specific CTLs were previously shown to lyse A*02:03 and A*02:06 BLCLs infected with LMP1-expressing vaccinia constructs^19^, while LMP2A_426-434_–specific CTLs similarly lysed LMP2A-expressing A*02:06 and A*02:07 BLCLs^31^. These peptides were predicted as non- or weak-binders of A*11:01 and A*24:02 (Supplementary Table S1). To test whether these EBV peptides could bind other HLA-A*02 subtypes, we performed pMHC stability ELISA and monitored the presence of β2 m as an indicator of effective peptide exchange and stability of the pMHC complex (Supplementary Figure S1)^25, 27^. All three EBV peptides were capable of replacing the conditional ligands and maintaining the structural integrity of the pMHC complexes, indicating that these peptides do bind A*02:03, A*02:06 and A*02:07 **(**Fig. 2).

**Table 1:** Binding prediction of EBV peptides EBNA1_562-570_, LMP1_125-133_, LMP2A_426-434_ to A*02:01, A*02:03, A*02:06 and A*02:07 haplotypes.

Peptide	HLA-A	Predicted affinity (nM)	Rank (%)	Bind Level
EBNA1_562-570_ FMVFLQTHI	02:01	21.27	0.3	++
	02:03	6.93	0.17	++
	02:06	28.81	0.6	+
	02:07	27279.25	4.5	−
LMP1_125-133_ YLLEMLWRL	02:01	2.13	0.01	++
	02:03	7.58	0.2	++
	02:06	2.84	0.01	++
	02:07	11737.13	0.6	+
LMP2A_426-434_ CLGGLLTMV	02:01	75.6	0.9	+
	02:03	6.79	0.17	++
	02:06	109.64	1.5	+
	02:07	7667.90	0.25	++

Prediction was performed by employing the online bioinformatics platform NetMHC (version 4.0) (http://www.cbs.dtu.dk/services/NetMHC-4.0/). Strong binders were indicated by a percentile rank (%rank) of less than 0.5, while weak binders ranged between 0.5 to less than 2. Values above 5 were considered as non-binders.

### TCR-like mAbs E1, L1 and L2 recognise EBV peptide-bound HLA-A*02 variants

**Figure 3:**
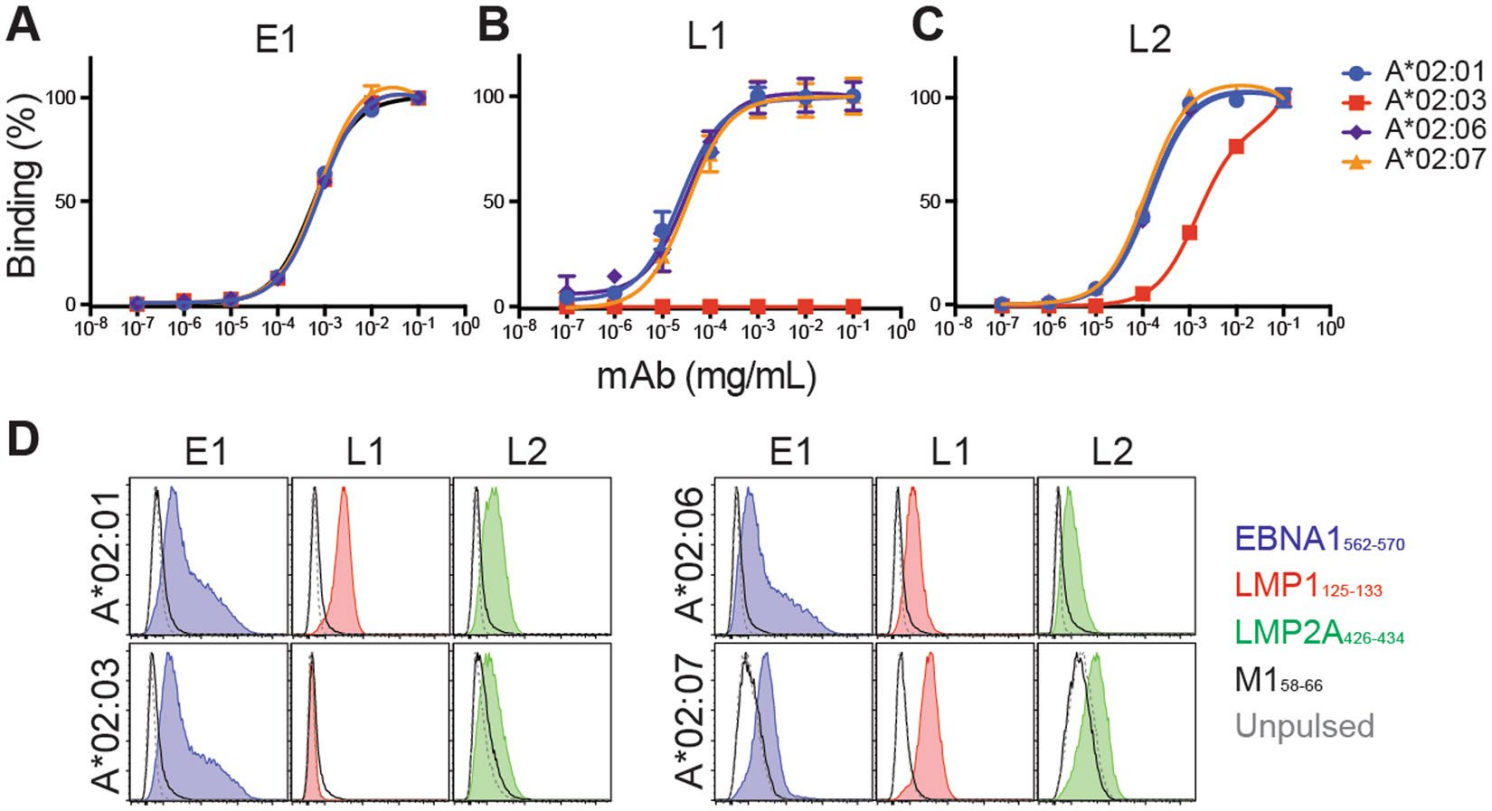
TCR-like mAbs E1, L1 and L2 bind EBV peptide-exchanged HLA-A*02 allelic variants. (**A**–**C**) Exchanged complexes of the respective peptides and HLA-A*02 molecules were added onto streptavidin-coated ELISA wells. TCR-like mAbs were 10-fold serially diluted and added into the wells before detection with HRP-conjugated secondary antibodies and readout at OD 415 nm (Supplementary Figure S1). Values were represented as percentage maximum binding and determined by non-linear regression graphs. (**D**) A*02:01, A*02:03, A*02:06 and A*02:07 EBV BLCLs were acid-stripped and pulsed with EBV EBNA1_562-570_ (blue), LMP1_125-133_ (red), LMP2A_426-434_ (green), *Influenza* M1_58-66_ (black), in addition to unpulsed control (grey dotted line). Shaded histograms depict the peptides pulsed and their corresponding staining for each TCR-like mAb.

To examine whether our anti-EBV TCR-like mAbs E1, L1 and L2 are able to bind EBV peptides in the context of other HLA-A*02 alleles, we performed ELISA using the peptide-exchanged pMHC complexes (Supplementary Figure S1). TCR-like mAbs displayed antibody concentration-dependent binding to their respective pMHC monomers, with E1 exhibiting similar binding profiles for all HLA-A*02 subtypes tested **(**Fig. 3A **)**. This was despite the indication that the EBNA1_562-570_ peptide might be less capable of stabilising the exchanged pMHC monomers for A*02:03, A*02:06 and A*02:07 **(**Fig. 2 **)**. Interestingly, there was a loss of binding between L1 and LMP1_125-133_–bound A*02:03 monomers **(**Fig. 3B **)**, as well as reduced binding between L2 and LMP2A_426-434_–bound A*02:03 monomers **(**Fig. 3C **)**. This might be attributed to the polymorphic residue substitutions at positions A149T, V152E and L156W found on top of the MHC molecule **(**Fig. 1D **)**, which might alter the binding interface between the antibodies and their recognition sites. Nevertheless, L1 and L2 recognised their respective peptides bound on A*02:06 and A*02:07 **(**Fig. 3B,C **)**. TCR-like mAbs similarly bound to their respective peptides exogenously pulsed on BLCLs expressing the four different HLA-A*02 variants, but not to those pulsed with the HLA-A*02-restricted *Influenza* M1_58-66_ control peptide **(**Fig. 3D **)**.

### E1, L1 and L2 bind endogenous targets on EBV BLCLs of HLA-A*02 variants

**Figure 4:**
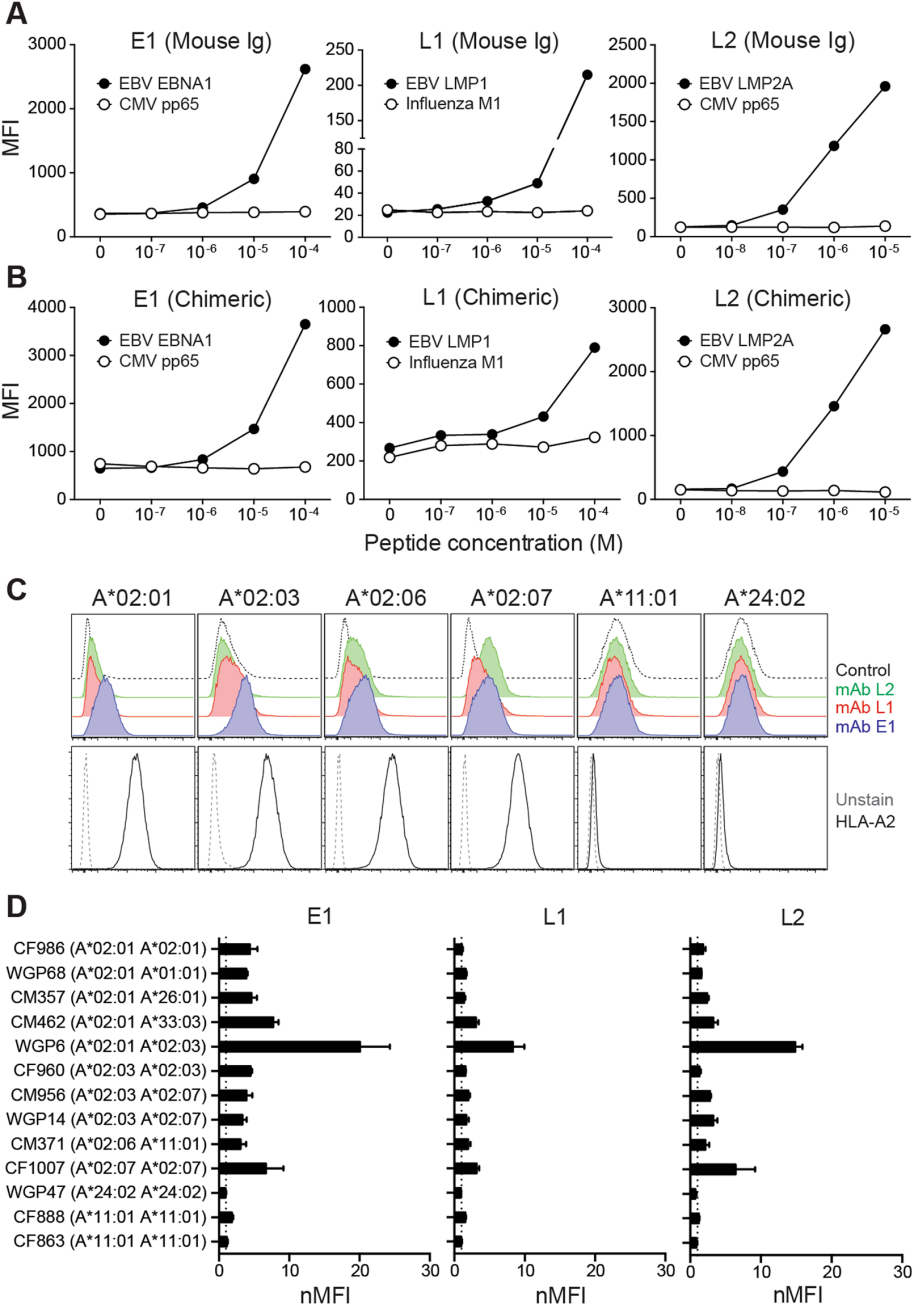
E1, L1 and L2 bind endogenous pMHC targets on EBV-transformed BLCLs. (**A**,**B**) T2 cells were pulsed with 10-fold serially diluted peptides before staining with (**A**) murine IgG1 or (**B**) chimerized IgG1 TCR-like mAbs. Cells were then stained with AlexaFluor-488 conjugated secondary antibodies for detection via flow cytometry. (**C**) Representative histogram plots of A*02:01, A*02:03, A*02:06 and A*02:07 EBV BLCLs stained with the respective chimeric TCR-like mAbs to detect endogenously presented pMHC surface targets. A*11:01 and A*24:02 BLCLs were used as controls. (**D**) Normalized mean fluorescence intensity (nMFI) of EBV BLCLs stained with the three TCR-like mAbs. Values were normalized by dividing the MFI reading from that of their respective secondary antibody control staining.

To evaluate the applicability of these antibodies beyond murine models, we chimerized the TCR-like mAbs using the human IgG1 framework for further analyses. Murine **(**Fig. 4A **)** and chimeric IgG1 **(**Fig. 4B **)** antibodies displayed similar peptide concentration-dependent binding trends on T2 cells pulsed with their respective peptides but not with the control peptides. As the density of pMHC complexes on pulsed cells does not physiologically reflect those found on infected cells, we tested the ability of these chimeric antibodies in recognising endogenously derived pMHC targets on EBV BLCLs. Chimeric E1, L1 and L2 mAbs showed different extents of binding to A*02:03, A*02:06 and A*02:07 EBV BLCLs, but not to those of A*11:01 or A*24:02 alleles **(**Fig. 4C **)**. This suggested that EBV BLCLs of closely related HLA alleles may process and present peptides in a similar manner. More importantly, HLA-A*02 micropolymorphisms do not strongly affect the binding of our TCR-like mAbs to these pMHC targets. As with our earlier studies, we again observed the highest staining profile with E1 antibody, followed by L2 and L1^7, 22^ **(**Fig. 4D **)**. Binding intensities of these TCR-like mAbs were independent of cell surface HLA-A*02 density, as indicated by staining with the HLA-A*02-specific BB7.2 antibody (Supplementary Figure S2).

### E1, L1 and L2 induce CDC against EBV BLCLs of HLA-A*02 variants

**Figure 5:**
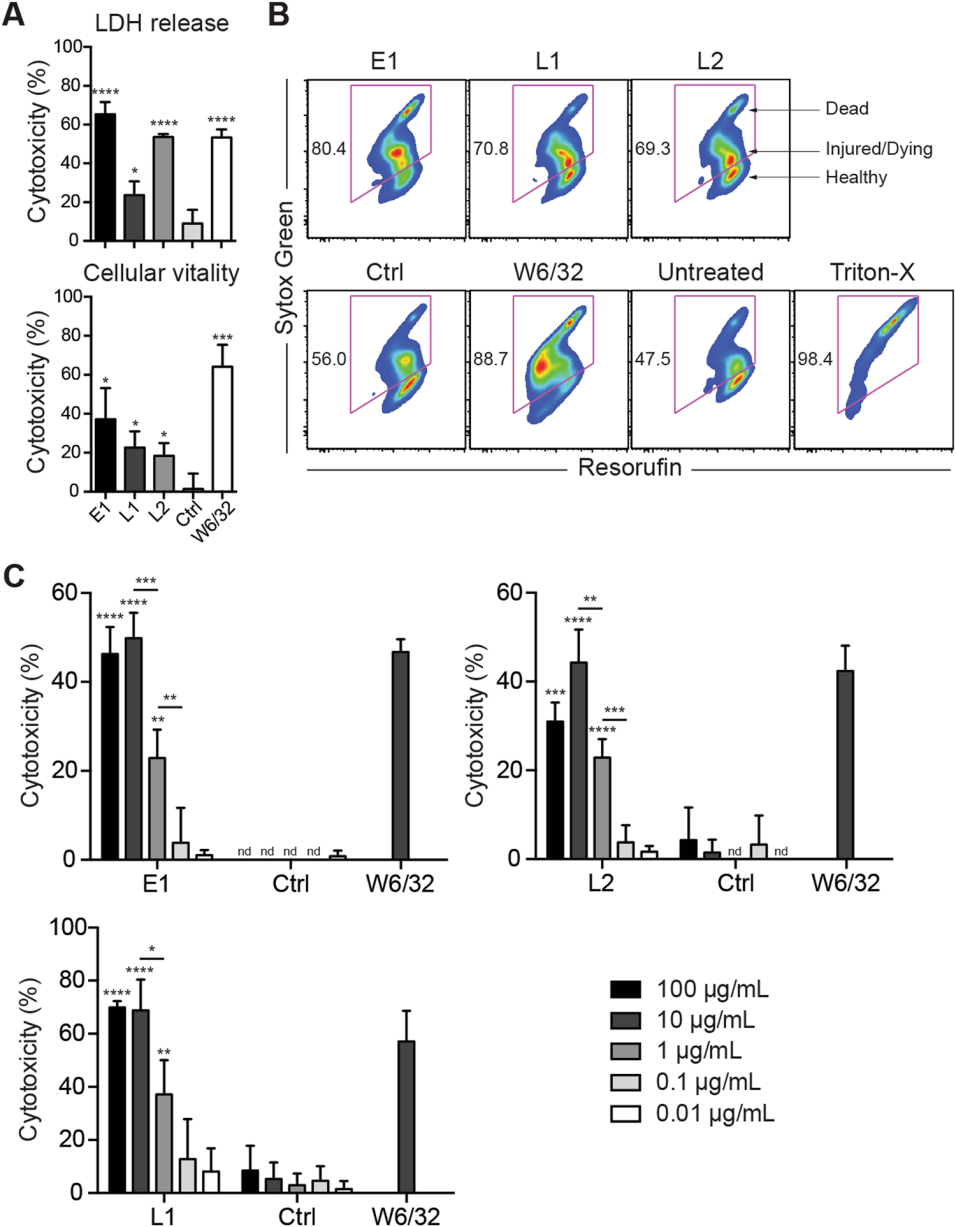
TCR-like mAbs mediate CDC against A*02:01 EBV BLCLs. (**A**) Homozygous A*02:01 BLCL CF986 was incubated with chimeric E1, L1, L2, murine IgG1 (MOPC-21, non-inducing negative control) or W6/32 (positive control) antibody (10 μg/mL) before addition of baby rabbit complement. Supernatants were assessed for LDH release, while cells were further processed for cellular vitality assay using C12-resaurzin and Sytox Green. (**B**) Representative flow cytometry plots for various antibody treatments in cellular vitality assay. **(C)** TCR-like mAbs were 10-fold serially diluted and incubated with A*02:01 A*02:03 BLCL WGP6 for CDC via LDH release. Values were expressed as mean ± SD, *p < 0.05, **p < 0.01, ***p < 0.001, ****p < 0.0001 (unpaired student’s t-test).

We previously reported the ability of our murine IgG1 TCR-like mAbs E1 and L2 in inducing early apoptosis and antibody-dependent phagocytosis of EBV BLCLs^7^. We therefore sought to determine whether the chimeric IgG1 format of these antibodies could elicit CDC on EBV BLCLs. As shown, chimeric E1, L1 and L2 mediated efficient CDC on homozygous A*02:01 BLCL CF986, as measured both by LDH release **(**Fig. 5A) and cellular vitality **(**Fig. 5A,B **)**. Furthermore, these antibodies displayed antibody-concentration dependent CDC on the highest expressing cell line tested, WGP6 (A*02:01 A*02:03), with significant cytolysis observed between 1–100 μg/mL of antibodies **(**Fig. 5C **)**. Notably, these antibodies could mediate cytolysis at percentages similar to the positive control pan-HLA murine IgG2a antibody W6/32, suggesting that these antibodies are effective inducers of CDC.

**Figure 6:**
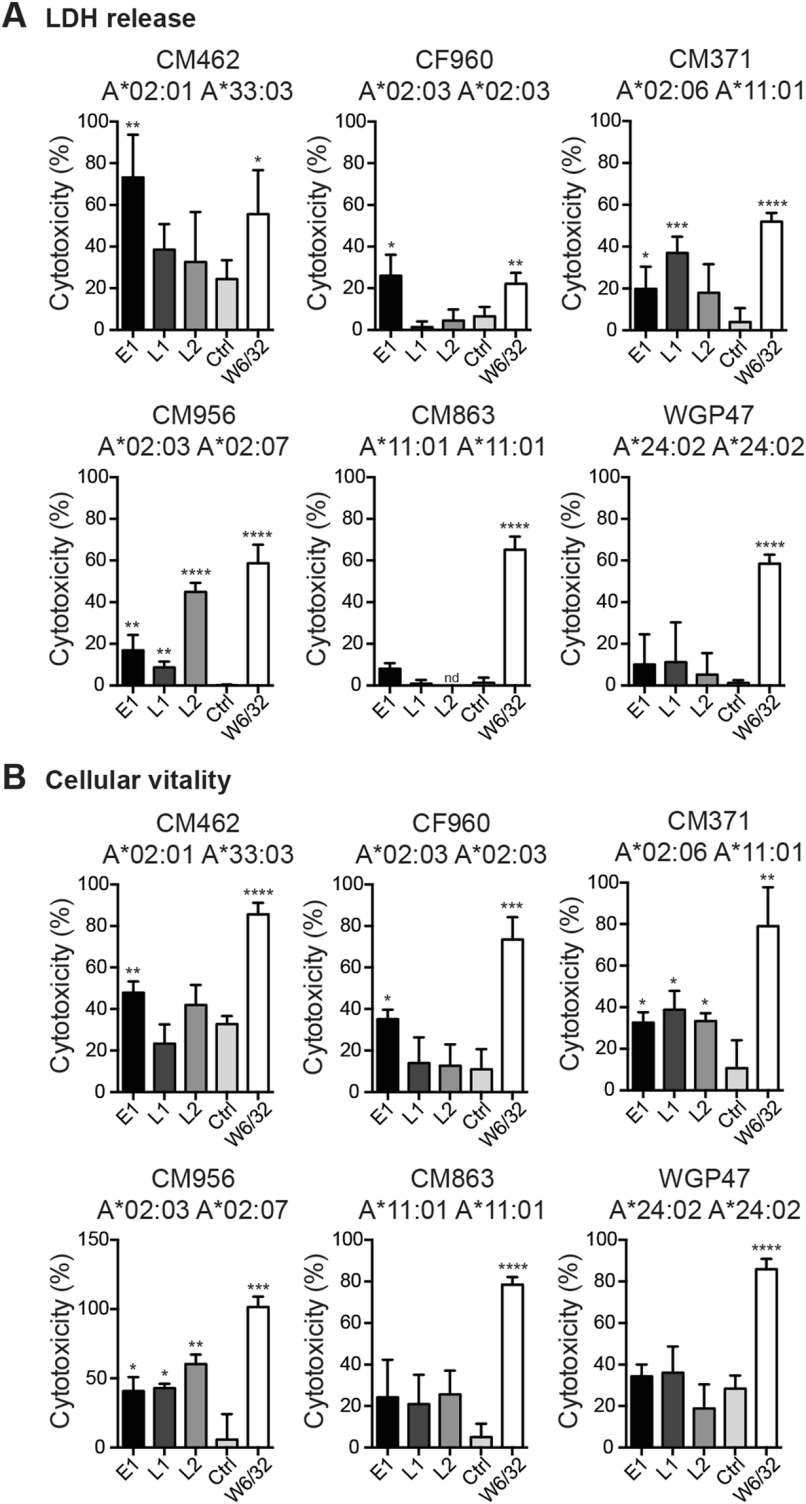
TCR-like mAbs mediate CDC against EBV BLCLs of HLA-A*02 variants. Six different EBV BLCLs were incubated with the respective antibodies (10 μg/mL) before the addition of baby rabbit complement and assessment of CDC by (**A**) LDH release and (**B**) cellular vitality. A*11:01 BLCL CM863 and A*24:02 BLCL WGP47 were included as control cell lines. Values were expressed as mean ± SD, *p < 0.05, **p < 0.01, ***p < 0.001, ****p < 0.0001 (unpaired student’s t-test).

We then tested whether these chimeric TCR-like mAbs could similarly mediate cytolysis against EBV BLCLs of other HLA-A*02 subtypes. E1, L1 and L2 induced CDC on heterozygous A*02:03 A*02:07 CM956 and A*02:06 CM371 EBV BLCLs **(**Fig. 6 **)**. As L1 and L2 mAbs displayed a loss of, or reduced binding towards A*02:03-bound EBV peptides **(**Fig. 3 **)**, it was likely that CM956 cytolysis was conferred through the recognition of A*02:07-presenting subtype. Indeed, E1 was the only antibody capable of mediating CDC against homozygous A*02:03 BLCL CF960. Interestingly, although heterozygous A*02:01 CM462 EBV BLCLs showed detectable staining with L1 and L2 **(**Fig. 4D **)**, these antibodies did not induce CDC against this cell line **(**Fig. 6 **)**. On the other hand, cytolysis was observed with CF960, CM956 and CM371 EBV BLCLs despite these cells having relatively lower surface antigen intensities than CM462 **(**Figs 4D and 6 **)**. This suggests that other intrinsic factors such as the expression of complement inhibitory receptors or sensitivity towards apoptosis may play a role in antibody-mediated CDC of tumor cells. As expected, anti-EBV TCR-like mAbs did not cause significant cytolysis against A*11:01 and A*24:02 control EBV BLCLs **(**Fig. 6 **)**, as well as EBV negative HLA-A2 positive B cell line BJAB (Supplementary Figure S3), indicating that antibody-induced CDC is both HLA- and EBV-specific.

### E1, L1 and L2 mediate ADCC of EBV BLCLs expressing HLA-A*02 allelic microvariants

**Figure 7:**
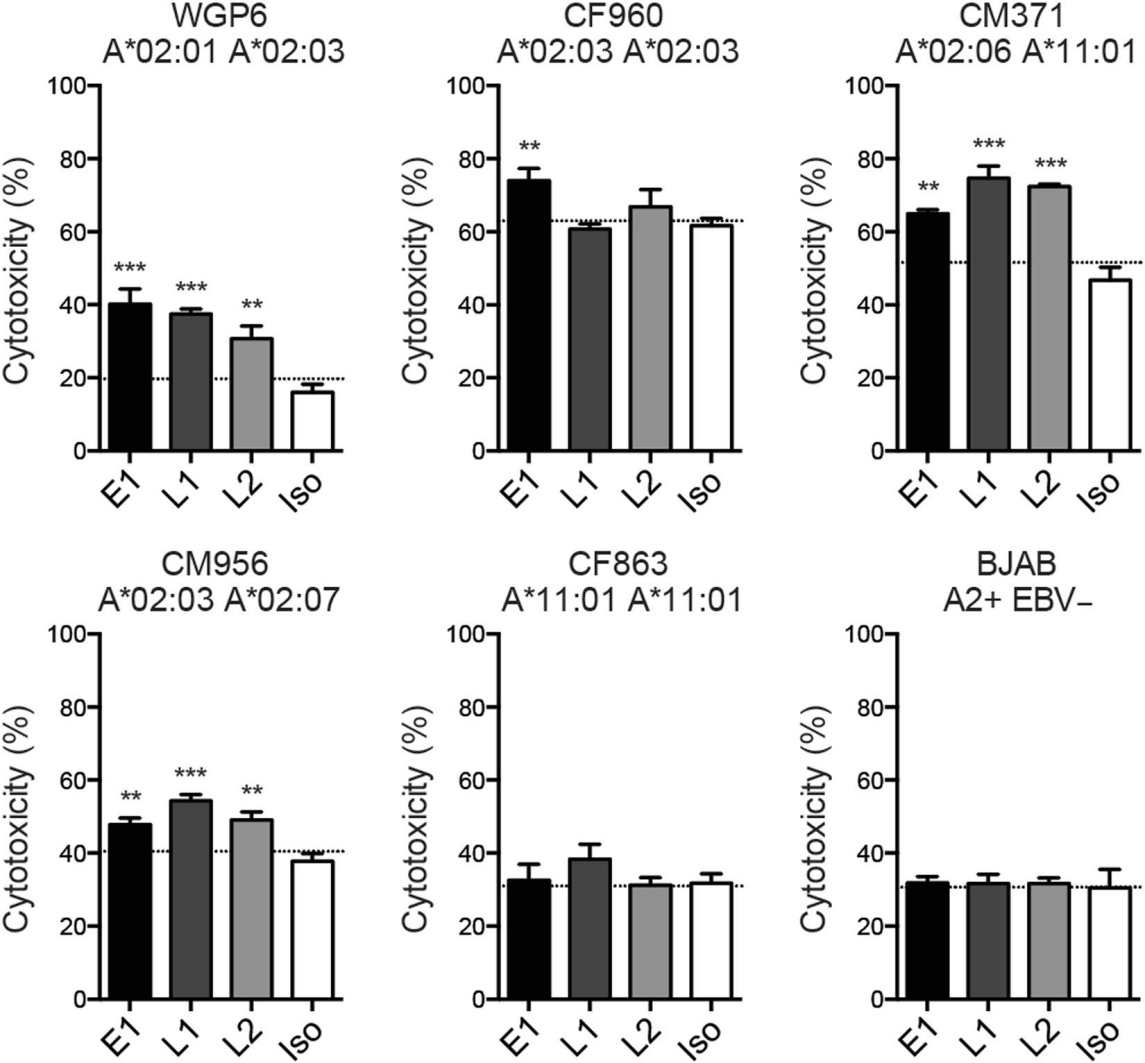
Anti-EBV TCR-like mAbs induce ADCC of BLCLs expressing HLA-A*02 subtypes. EBV BLCLs were labeled with CFSE and stained with the respective chimeric E1, L1, L2 or isotype human IgG1 control antibody prior to co-culturing with freshly isolated PBMCs for 4 hours (E:T 20:1). Samples were assessed via flow cytometry to determine the percentage of CFSE + 7-AAD + cells. Dotted line denotes the level of spontaneous release with no antibody treatment. Values were expressed as mean ± SD, *p < 0.05, **p < 0.01, ***p < 0.001 (unpaired student’s t-test).

As one of the major antibody effector functions against tumor cells^2^, we next determined whether our chimeric anti-EBV TCR-like mAbs could induce ADCC of EBV BLCLs. CFSE-labeled BLCLs were incubated with the respective antibodies plus PBMCs, and ADCC was evaluated via flow cytometry^32^ (Supplementary Figure S4). E1, L1 and L2 exhibited significantly enhanced cellular cytotoxicity against EBV BLCLs of various HLA-A*02 subtypes including A*02:01 A*02:03 WGP6, A*02:06 CM371 and A*02:03 A*02:07 CM956. Similar to the CDC assays, E1 but not L1 or L2 was able to induce ADCC of homozygous A*02:03 CF960, and neither of these antibodies displayed non-specific killing of A*11:01 CF863 or A2+EBV− BJAB cell lines **(**Fig. 7 **)**. Taken together, these data suggest that the chimeric formats of the TCR-like mAbs are potent inducers of CDC and ADCC against EBV BLCLs expressing different HLA-A*02 variants.

Notably, the ability of E1 to mediate cytolytic activity against the majority of EBV BLCLs tested is in agreement with the high expression of its presented target antigen across heterogeneous BLCLs^7, 22^. This may suggest that surface epitope density is an important indicator of the effectiveness of antibody-targeted killing. While L1 and L2 revealed generally lower staining profiles than E1, several approaches may potentially improve the intensities of their surface target antigens. This is a critical aspect in TCR-like mAb therapy, as pMHC complexes are often present at levels lower than those targeted by conventional antibodies. The pharmacological modulation of HLA expression using cytokines may possibly enhance L1- and L2-mediated killing of EBV BLCLs. As with other TCR recognition-based strategies, additional studies would also be required to evaluate any potential off-target recognition of these antibodies^33, 34^. Nevertheless, our data demonstrated that anti-EBV TCR-like mAbs E1, L1 and L2, which were originally raised in the context of A*02:01, were able to perform antibody effector functions against EBV BLCLs of closely related HLA-A*02 variants. This implies that for populations with a huge diversity in HLA-A*02 microvariants, such as those in Southeast Asia, the prospective coverage of such reagents may potentially increase from approximately 30% to more than 90% of HLA-A*02 individuals. Indeed, ongoing clinical trials (NCT01343043, NCT02588612) involving genetically engineered T cells that target an A*02:01-restricted NY-ESO-1 peptide (NY-ESO-1^c259^T) have in the recent years expanded their eligibility criteria to include A*02:05 and A*02:06 patients. Our findings suggest that peptide binding degeneracy may be explored and possibly exploited with respect to TCR-like mAbs and TCR recognition-based therapies, and that such approaches may be expanded to include individuals expressing different HLA-A*02 allelic variants.

## Electronic supplementary material

Supplementary Information

## References

[1] Dahan R, Reiter Y (2012). T-cell-receptor-like antibodies - generation, function and applications. Expert Rev Mol Med.

[2] Dao T (2013). Targeting the Intracellular WT1 Oncogene Product with a Therapeutic Human Antibody. Science Translational Medicine.

[3] Jain R, Rawat A, Verma B, Markiewski MM, Weidanz JA (2013). Antitumor activity of a monoclonal antibody targeting major histocompatibility complex class I-Her2 peptide complexes. J. Natl. Cancer Inst..

[4] Sastry KSR (2011). Targeting hepatitis B virus-infected cells with a T-cell receptor-like antibody. J Virol.

[5] Verma B (2011). TCR mimic monoclonal antibodies induce apoptosis of tumor cells via immune effector-independent mechanisms. The Journal of Immunology.

[6] Sergeeva A (2011). An anti-PR1/HLA-A2 T-cell receptor-like antibody mediates complement-dependent cytotoxicity against acute myeloid leukemia progenitor cells. Blood.

[7] Lai, J. *et al*. Targeting Epstein-Barr virus transformed B lymphoblastoid cells using antibodies with T cell receptor-like specificities. *Blood*, doi:10.1182/blood-2016-03-707836 (2016). 10.1182/blood-2016-03-707836 PMC5054486 27338099

[8] Burrows SR, Miles JJ (2013). Immune parameters to consider when choosing T-cell receptors for therapy. Front Immunol.

[9] Sommer S (2005). The importance of immune gene variability (MHC) in evolutionary ecology and conservation. Front. Zool..

[10] Krausa P (1995). Genetic polymorphism within HLA-A*02: significant allelic variation revealed in different populations. Tissue Antigens.

[11] Browning M, Krausa P (1996). Genetic diversity of HLA-A2: evolutionary and functional significance. Immunol. Today.

[12] Doolan DL (1997). Degenerate cytotoxic T cell epitopes from P. falciparum restricted by multiple HLA-A and HLA-B supertype alleles. Immunity.

[13] Threlkeld SC (1997). Degenerate and promiscuous recognition by CTL of peptides presented by the MHC class I A3-like superfamily: implications for vaccine development. J. Immunol..

[14] Burrows SR (2003). Promiscuous CTL recognition of viral epitopes on multiple human leukocyte antigens: biological validation of the proposed HLA A24 supertype. J. Immunol..

[15] Kondo E (2004). Identification of novel CTL epitopes of CMV-pp65 presented by a variety of HLA alleles. Blood.

[16] Choo JAL, Liu J, Toh X, Grotenbreg GM, Ren EC (2014). The immunodominant influenza A virus M158-66 cytotoxic T lymphocyte epitope exhibits degenerate class I major histocompatibility complex restriction in humans. J Virol.

[17] Barouch D (1995). HLA-A2 subtypes are functionally distinct in peptide binding and presentation. J. Exp. Med..

[18] Sidney J (2001). Majority of peptides binding HLA-A*0201 with high affinity crossreact with other A2-supertype molecules. Hum. Immunol..

[19] Khanna R, Burrows SR, Nicholls J, Poulsen LM (1998). Identification of cytotoxic T cell epitopes within Epstein-Barr virus (EBV) oncogene latent membrane protein 1 (LMP1): evidence for HLA A2 supertype-restricted immune recognition of EBV-infected cells by LMP1-specific cytotoxic T lymphocytes. Eur. J. Immunol..

[20] Zhang H-G, Pang X-W, Shang X-Y, Xing Q, Chen W-F (2003). Functional supertype of HLA-A2 in the presentation of Flu matrix p58-66 to induce CD8+ T-cell response in a Northern Chinese population. Tissue Antigens.

[21] Young LS, Rickinson AB (2004). Epstein-Barr virus: 40 years on. Nat Rev Cancer.

[22] Sim ACN (2013). Defining the expression hierarchy of latent T-cell epitopes in Epstein-Barr virus infection with TCR-like antibodies. Sci Rep.

[23] Nielsen M (2003). Reliable prediction of T-cell epitopes using neural networks with novel sequence representations. Protein Sci..

[24] Andreatta M, Nielsen M (2016). Gapped sequence alignment using artificial neural networks: application to the MHC class I system. Bioinformatics.

[25] Chang CXL (2013). Conditional ligands for Asian HLA variants facilitate the definition of CD8+ T-cell responses in acute and chronic viral diseases. Eur. J. Immunol..

[26] Choo JAL (2014). Bioorthogonal cleavage and exchange of major histocompatibility complex ligands by employing azobenzene-containing peptides. Angew. Chem. Int. Ed. Engl..

[27] Toebes M (2006). Design and use of conditional MHC class I ligands. Nat Med.

[28] Stuber G (1995). HLA-A0201 and HLA-B7 binding peptides in the EBV-encoded EBNA-1, EBNA-2 and BZLF-1 proteins detected in the MHC class I stabilization assay. Low proportion of binding motifs for several HLA class I alleles in EBNA-1. Int. Immunol..

[29] Lee SP (1993). HLA A2.1-restricted cytotoxic T cells recognizing a range of Epstein-Barr virus isolates through a defined epitope in latent membrane protein LMP2. J Virol.

[30] Sette A, Sidney J (1999). Nine major HLA class I supertypes account for the vast preponderance of HLA-A and -B polymorphism. Immunogenetics.

[31] Lee SP, Tierney RJ, Thomas WA, Brooks JM, Rickinson AB (1997). Conserved CTL epitopes within EBV latent membrane protein 2: a potential target for CTL-based tumor therapy. J. Immunol..

[32] Salinas-Jazmín N, Hisaki-Itaya E, Velasco-Velázquez MA (2014). A flow cytometry-based assay for the evaluation of antibody-dependent cell-mediated cytotoxicity (ADCC) in cancer cells. Methods Mol Biol.

[33] Chinnasamy N (2011). A TCR targeting the HLA-A*0201-restricted epitope of MAGE-A3 recognizes multiple epitopes of the MAGE-A antigen superfamily in several types of cancer. The Journal of Immunology.

[34] Morgan RA (2013). Cancer regression and neurological toxicity following anti-MAGE-A3 TCR gene therapy. J. Immunother..

