# Supplementary material for: TCR–like antibodies mediate complement and antibody-dependent cellular cytotoxicity against Epstein-Barr virus–transformed B lymphoblastoid cells expressing different HLA-A*02 microvariants
Source: Sci Rep. 2017 Aug 30;7:9923. doi: 10.1038/s41598-017-10265-6 (PMC5577143; doi:10.1038/s41598-017-10265-6)

TCR–like antibodies mediate complement and antibody-dependent cellular cytotoxicity against Epstein-Barr virus–transformed B lymphoblastoid cells expressing different HLA-A*02 microvariants

Junyun Lai, Joanna Ai Ling Choo, Wei Jian Tan, Chien Tei Too, Min Zin Oo, Manuel A. Suter, Fatimah Bte Mustafa, Nalini Srinivasan, Conrad En Zuo Chan, Andrew Guo Xian Lim, Youjia Zhong, Soh Ha Chan, Brendon J. Hanson, Nicholas R. J. Gascoigne and Paul A. MacAry

Supplementary Information

Supplementary Figure Legends

Supplementary Figure S1. Schematic illustration of pMHC ELISA. In this conditional ligand exchange technology, a UV-sensitive cognate peptide that is embedded on a desired MHC molecule is cleaved in the presence of a peptide in question. Peptides that bind to the MHC molecule will stabilise the pMHC complex and prevent the dissociation of the β2m subunit, whereas a non-binding peptide will result in the disintegration of the complex, thereby releasing the β2m subunit. The presence of the non-covalently bound β2m serves as an indication of peptide binding in pMHC stability ELISA (left). For TCR-like mAb pMHC ELISA (right), exchanged pMHC complexes are further incubated with the antibodies and binding is detected using a HRP-conjugated secondary antibody.

Supplementary Figure S2. Normalized MFI of HLA-A2 expression on EBV BLCLs. HLA-A*02 expression was assessed using murine BB7.2 antibody. MFI were normalized by dividing the values from that of their respective unstained control readings.

Supplementary Figure S3. LDH and cellular vitality profiles of HLA-A2 positive EBV negative BJAB under CDC assay. BJAB cells were incubated with chimeric E1, L1, L2, murine IgG1 (negative control) or W6/32 (positive control) antibody (10 μg/mL) before addition of baby rabbit complement. Supernatants and cells were respectively assessed for LDH release and cellular vitality using C12-resaurzin and Sytox Green. Values were expressed as mean ± SD, *$p<0.05$, ****$p<0.0001$ (unpaired student's t-test).

Supplementary Figure S4. Gating strategy for analysis of ADCC. Samples were gated based on forward versus side scatter plot to exclude cellular debris and doublets. Target cells were then further gated based on CFSE-labeling, and the percentages of 7-AAD+ cells were evaluated accordingly.

Supplementary Table Legend

Supplementary Table 1. Binding prediction of EBV peptides to A*11:01 and A*24:02 haplotypes. Prediction was performed through NetMHC (version 4.0). Percentile rank values between 0.5 to less than 2 were considered as weak binders, while those above 5 were indicated as non-binders.

Supplementary Table S1

Peptide	HLA-A	Predicted affinity (nM)	Rank (%)	Bind Level
$EBNA1_{562-570}$	11:01	29789.5	32.0	-
FMVFLQTHI	24:02	10125.3	6.0	-
$LMP1_{125-133}$	11:01	24570.1	22.0	-
YLLEMLWRL	24:02	1196.7	1.3	+
$LMP2A_{426-434}$	11:01	30206.9	33.0	-
CLGGLLTMV	24:02	24210.7	17.0	-

Supplementary Figure S1

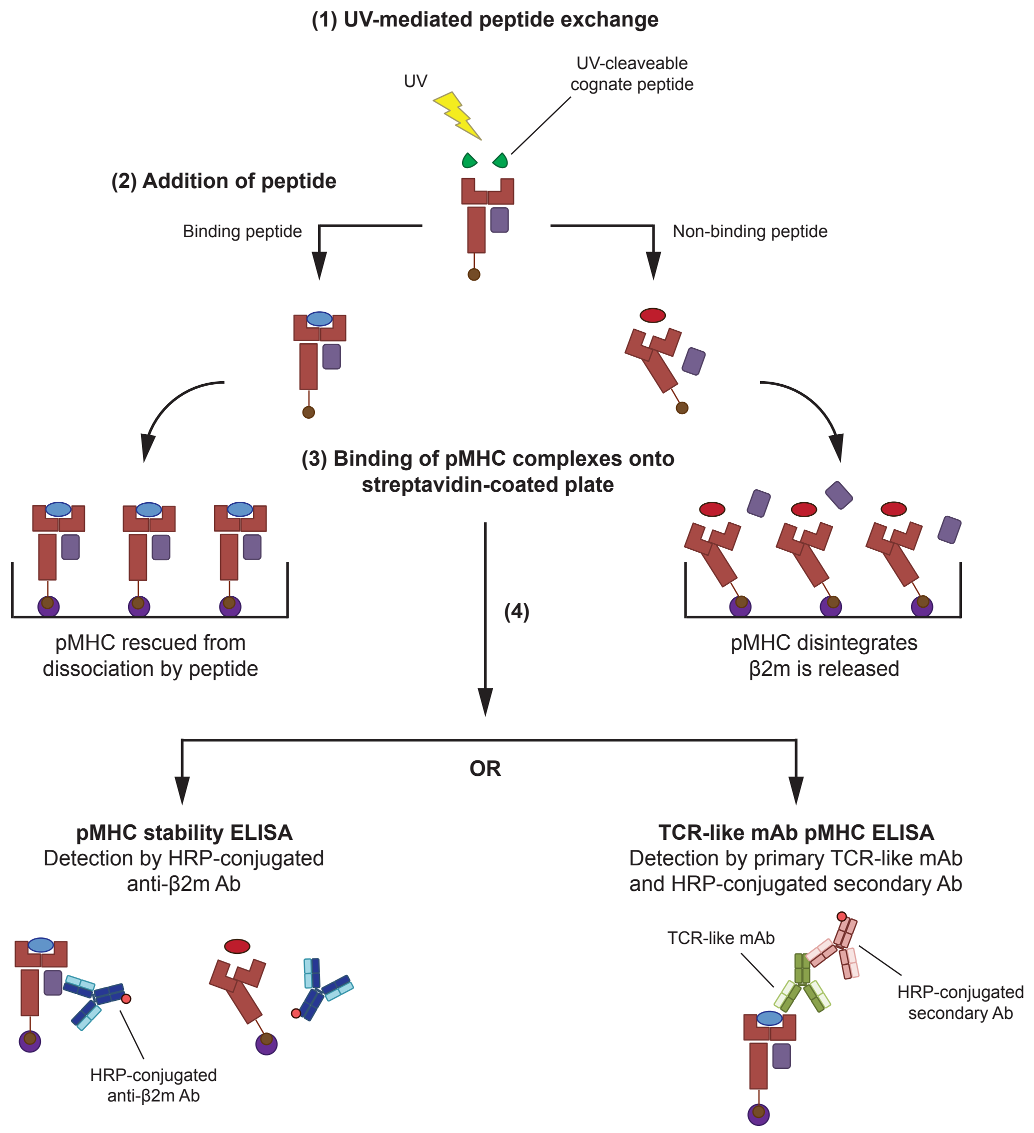

Supplementary Figure S2

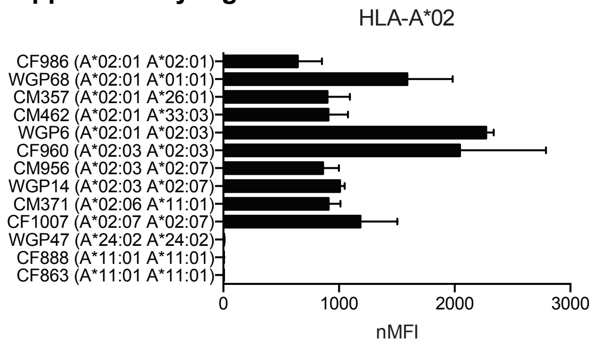

Supplementary Figure S3

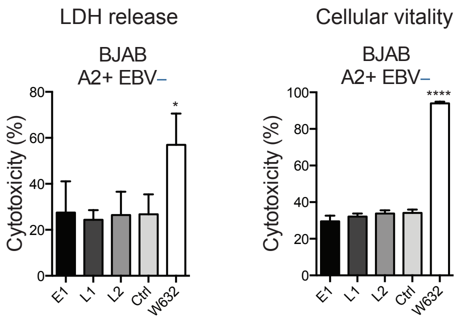

Supplementary Figure S4

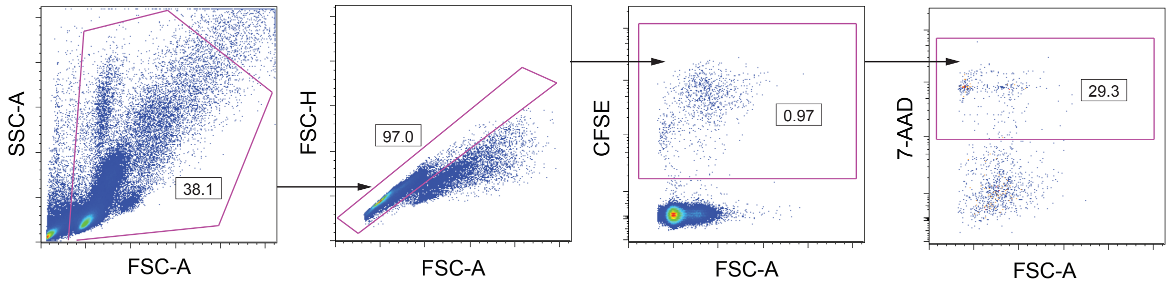

Supplement: Supplementary file 1 — Supplementary Information [file 41598_2017_10265_MOESM1_ESM.pdf]
